# Advances in Physiologically Based Pharmacokinetic (PBPK) Modeling and its Regulatory Utility to Support Oral Drug Product Development and Harmonization

**DOI:** 10.1007/s11095-025-03849-9

**Published:** 2025-03-28

**Authors:** Yi-Hsien Cheng, Sherin Thomas, Yu Chung Tsang, Susana Almeida, Muhammad Ashraf, Nikoletta Fotaki, Tycho Heimbach, Nikunjkumar Patel, Harshil Shah, Xiaojian Jiang, Myong-Jin Kim, Rebecca Moody, Amin Rostami‐Hodjegan, Romi Singh, Liang Zhao, Andrew Babiskin, Fang Wu

**Affiliations:** 1 Office of Research and Standards (ORS), Office of Generic Drugs (OGD), Center for Drug Evaluation and Research (CDER), U.S. Food and Drug Administration (FDA), Silver Spring, Maryland USA; 2 YCT Scientific Inc., Toronto, Canada; 3 International Generic and Biosimilar Medicines Association, Geneva, Switzerland; 4 Office of Pharmaceutical Quality Research (OPQR), Office of Pharmaceutical Quality (OPQ), CDER, U.S. FDA, Silver Spring, Maryland USA; 5 Centre for Therapeutic Innovation, Department of Life Sciences, University of Bath, Claverton Down, Bath, BA2 7AY UK; 6 Pharmaceutical Sciences and Clinical Supply, Merck & Co., Inc, Rahway, New Jersey USA; 7 Certara Predictive Technologies Division, Sheffield, UK; 8 Cosette Pharmaceuticals Inc., New Jersey, USA; 9 Office of Bioequivalence (OB), OGD, CDER, U.S. FDA, Silver Spring, Maryland USA; 10 Office of Product Quality Assessment II (OPQA II), OPQ, CDER, U.S. FDA, Silver Spring, Maryland USA; 11 Centre for Applied Pharmacokinetic Research (CAPKR), University of Manchester, Manchester, UK; 12 Formulation Research and Development - Orals, Sun Pharmaceuticals Industries Ltd., Gurugram, India

**Keywords:** harmonization, physiologically based pharmacokinetic (PBPK) absorption modeling, virtual bioequivalence (VBE)

## Abstract

This report summarizes the proceedings of Session 1 of the one-day public workshop titled “*Advances in PBPK Modeling and its Regulatory Utility for Oral Drug Product Development*” hosted by the U.S. Food and Drug Administration (FDA) and the Center for Research on Complex Generics (CRCG) on October 12, 2023. This report focuses on cutting-edge developments, ongoing challenges, and potential solutions in the field of physiologically based pharmacokinetic (PBPK) absorption modeling for systemic and gastrointestinal (GI) locally acting oral drug products, as well as exploring opportunities to enhance global harmonization for generic drug development. Despite significant advancements and several successful case studies of utilizing PBPK models in generic drug development, developing patient-centric dissolution quality standards using PBPK modeling that account for food effects or different disease states remains challenging. Combining multiple dissolution studies at different pH ranges can aid in developing patient-centric dissolution specifications. Additionally, a major challenge for GI locally acting drug products is the inability to validate the PBPK model for local bioavailability due to the lack of measured data for local drug concentration along the different sections of the GI tract. A totality of evidence-based approach, taking account of all available data in addition to PBPK modeling-based evidence, should be considered. Moving forward, it is crucial to promote global collaboration and research by sharing knowledge and experiences for utilizing PBPK models in regulatory contexts to advance both internal and international harmonization.

## Introduction

The U.S. Food and Drug Administration (FDA) and the Center for Research on Complex Generics (CRCG) held a workshop titled “*Advances in PBPK Modeling and its Regulatory Utility for Oral Drug Product Development*” on October 12, 2023. This workshop engaged experts from the FDA, the brand and generic drug industries, consultants, academia, and other stakeholders in the area of modeling and simulation (M&S). The purpose of this one-day workshop was to discuss the challenges, experiences, and advances related to the development of oral physiologically based pharmacokinetic (PBPK) absorption modeling. This modeling supports the establishment of biopredictive in vitro testing and addresses risks associated with the extrapolation of bioequivalence (BE) studies in various contexts. Biopredictive dissolution method is a set of testing conditions for which in vitro dissolution profiles are capable of predicting PK profiles, whereas biorelevant dissolution method is designed for in vitro dissolution that can closely mimic a relevant biological fluid and a physiological environment [1]. This workshop provided case examples for extrapolating from fasting to fed states, extrapolating from subjects with normal pH to those with elevated gastric pH, justifying BE study designs, supporting Biopharmaceutics Classification System (BCS)-based biowaivers, assessing BE in pediatrics, and conducting other risk-based BE assessments for oral drug products. Recently, there were published manuscripts providing comprehensive summaries of best practices for incorporating drug product quality attributes within physiologically based biopharmaceutics modeling (PBBM) to support product development programs and regulatory submissions [2–5]; this workshop focused on regulatory utilities of PBPK modeling for BE assessment.

In particular, Session 1, titled "*Advances in PBPK Modeling in Regulatory Contexts and to Support Harmonization*" delved into the cutting-edge developments, challenges, and potential solutions for employing PBPK absorption modeling and virtual BE (VBE) simulation in regulatory submissions. This session highlighted the pivotal role these models play in supporting oral drug product development, conducting risk-based assessments, and addressing regulatory questions. Additionally, it explored the opportunities for enhancing global harmonization in regulatory practices, such as comparison and interpretation of recently released guidance, “International Council for Harmonisation of Technical Requirements for Pharmaceuticals for Human Use (ICH) M13A: Bioequivalence for Immediate-Release Solid Oral Dosage Forms” [6] and “Guidance for Industry: Bioequivalence Studies with Pharmacokinetic Endpoints for Drugs Submitted under an Abbreviated New Drug Application (ANDA)” [7]. The session covered critical considerations for the development and validation of PBPK models aimed at predicting both systemic and local drug exposure of oral drug products. It emphasized the importance of accurately modeling gastrointestinal (GI) locally acting drug products, particularly in distinguishing between healthy and diseased populations. Experts presented and discussed key aspects about implications of PBPK modeling for streamlining regulatory pathways, reducing the need for extensive in vivo testing, and facilitating more efficient drug development processes.

Specifically, a well-established and sufficiently validated PBPK absorption model, also recently referred to as PBBM, effectively addresses regulatory questions and supports decision-making by providing a robust framework for various applications [2, 8–12]. These include justifying BE study designs, such as comparing fasting vs. fed conditions for drug administration or determining the analytes for BE assessment from the parent drug vs. metabolites. The model can assess the risks associated with formulation changes on drug absorption and overall pharmacokinetics, evaluate the impact of non-comparable in vitro dissolution results on systemic exposure and BE determination (e.g., in vitro alcohol dose dumping (ADD) studies), and support BE study designs tailored for specific populations (e.g., single-sex vs. both sexes). Additionally, it can provide evidence to justify waivers for certain in vivo studies based on M&S results. Since conventional in vitro dissolution testing often poorly correlates with pharmacokinetic (PK) data in patients, a well-developed PBPK model that incorporates biorelevant/biopredictive in vitro dissolution data as model inputs can adequately predict systemic drug exposures in both healthy individuals and those with different disease states, establish clinically relevant dissolution specifications, and develop patient-centric dissolution quality standards. This approach helps create drug products that better meet patients' needs, leading to improved therapeutic outcomes and enhanced patient satisfaction.

For GI locally acting drug products, demonstrating BE is challenging because these drug products reach the site of action before they enter systemic circulation. Systemic exposure may not reflect drug concentrations at the site of action because drug absorption can take place from different regions of GI tract. BE recommendations of GI locally acting oral drug products are based on drug product properties and mechanism of action. In addition, PBPK models may be used for exploring the correlation between dissolution in the GI tract and plasma concentration profiles, identifying biopredictive dissolution and predicting local drug concentration/amount and thus, for supporting BE evaluation of such products. Totality of evidence based-approach based on dissolution data, formulation differences, gut physiology considerations along with PBPK modeling results can be used to conduct BE-based risk assessment for these products.

The following sections provide highlights from podium presentations, panel discussions, and roundtable deliberations. The workshop agenda, speaker bios, presentation slides, and recordings can be found at CRCG website [13].

## Podium Presentations

### Summary of Presentation “Advances on Using PBPK Modeling to Support BE Assessment for Oral Products” by Dr. Fang Wu (Senior Pharmacologist in Division of Quantitative Methods and Modeling (DQMM), Office of Research and Standards (ORS), Office of Generic Drugs (OGD) of the FDA)

**Fig. 1:**
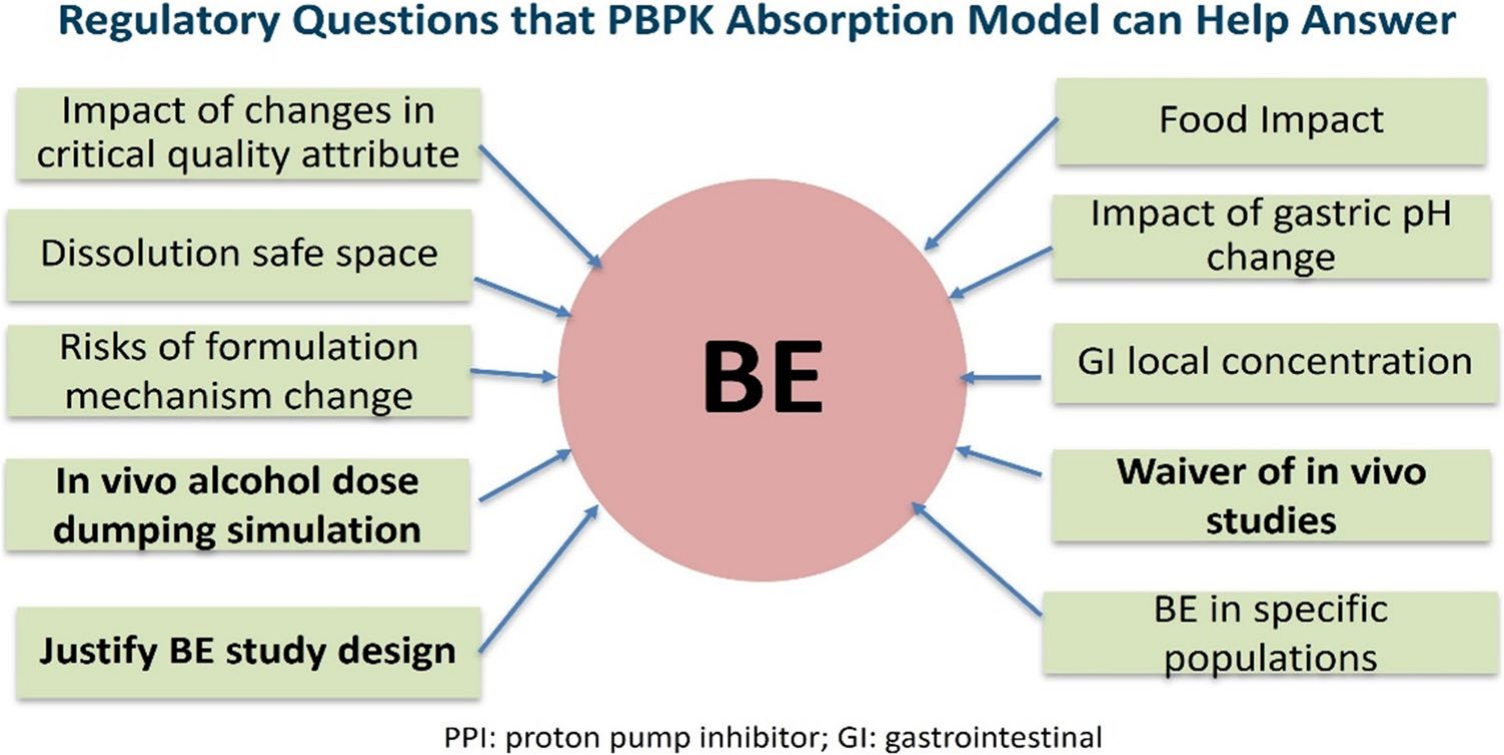
Regulatory questions that physiologically based pharmacokinetic (PBPK) absorption model can help answer in generic drug development with three highlighted applications presented.

Dr. Wu presented the recent advances and regulatory case examples of PBPK modeling for oral products (see highlighted applications in Fig. 1), as well as the utilities of PBPK absorption models mentioned in ICH M13A draft guideline for the evaluation of generic drugs within OGD [6]. First, Dr. Wu presented a case example about using PBPK modeling to evaluate the impact of in vitro ADD on BE determination for an extended-release (ER) capsule drug product. It was observed that there was increased release from the 200 mg Test product compared to the reference listed drug (RLD) with 20% ethanol in the in vitro ADD study. No difference in the in vitro release between Test and RLD was observed when the ADD study was conducted with 5% or 40% ethanol. A PBPK absorption model was developed to evaluate whether the increased drug release of Test product would significantly impact the systemic exposure compared to the RLD which has lower release. The model predictions were compared against the observed PK data of RLD by incorporating representative dissolution data at pH 1.1, pH 7.5, and bi-phasic dissolution. The bi-phasic dissolution profiles were constructed from dissolution at pH 1.1 for 1 hour combined with dissolution at pH 7.5 after 1 hour. Dissolution at pH 1.1 was found not to be biopredictive whereas the bi-phasic dissolution was biopredictive to the PK profiles of RLD as the developed bi-phasic dissolution profile appears to better mimic real in vivo drug release. Then, VBE simulations were conducted with inputs of bi-phasic dissolution with 20% ethanol. The simulation results suggested that the increased drug release of the Test product at pH 1.1 with 20% ethanol will not result in significant differences in systemic exposure, compared to the RLD product. Hence, it was concluded that there is no safety concern arising from the Test product with higher release in the in vitro ADD study.

The second case example utilizing PBPK modeling was to evaluate the impact of sex on BE assessment for atorvastatin tablets to justify BE study design. Per the RLD labeling [14], females have 20% higher Cmax and 10% lower AUC, as compared to males. The applicant conducted BE studies using male-only subjects. The applicant submitted atorvastatin PBPK model to support the justification that BE results from male-only subjects can be extrapolated to the entire population (i.e., both sexes). However, limitations were identified on the model submitted by the applicant. For example, the model was not properly developed to capture the sex differences in drug exposure. In addition, the applicant did not conduct VBE simulations using properly developed and sufficiently validated PBPK model to support its justification of using male-only population for BE assessment. Hence, during FDA assessment, the model was optimized to capture the trend of sex differences in drug exposure, i.e., females have higher Cmax and lower AUC compared to males. The PBPK model that can predict adequately the trend of sex differences in drug exposure was used to evaluate BE for different populations (0%, 50%, and 100% female subjects included in each trial) in VBE simulations. The VBE simulation results showed that with different percentages of female subjects, the 90% CI of Cmax and AUC ratio between Test and RLD products are all within 80 to 125%. This supported that the risk of having different BE results is low by using male-only subjects, compared to using subjects of both sexes. It is worth mentioning that the default variabilities in the GI tract attributes, which appear to be the same between males and females, were used in the simulations. Generally, intrasubject variabilities were similar between males and females [15].

In addition, regulatory research showed that PBPK modeling approach may also be used together with in vitro studies (e.g., permeation testing) to evaluate the impact of excipients (including antioxidant) on bioavailability (BA) and/or BE of new and generic drug products and support formulation development to address impurity and reformulation issue. Besides the above-mentioned case examples, the utilities of PBPK modeling were also mentioned in recently issued ICH M13A guideline in January 2023 [6]. That is, appropriately validated PBPK modeling may be used to support the rationale for the design of a BE study with regard to the use of fasting and/or fed conditions. Modeling can also be used to assess whether the generic drug and RLD are still BE in subjects with elevated pH and to support waiving of relevant BE studies.

### Summary of Presentation “PBPK Absorption Modeling for Developing Patient‐Centric Quality Standards” by Dr. Muhammad Ashraf (Supervisor for Division of Pharmaceutical Quality Research V (DPQRV), Office of Pharmaceutical Quality Research (OPQR), Office of Pharmaceutical Quality (OPQ) of the FDA)

Dr. Ashraf underscored the critical role of patient-centric quality standards (PCQS) in confirming acceptable in vivo performance of drug products, emphasizing the potential imprecision in establishing PCQS based solely on an empirical relationship between in vitro dissolution and in vivo performance, especially when not accounting for biological conditions of drug absorption in the GI tract and physicochemical characteristics of the drug substances and the formulation excipients and design.

The presentation further addressed recent regulatory communications questioning the clinical relevance of dissolution testing, advocating for further research in this domain. The gap identified in understanding critical parameters for developing biorelevant dissolution methods and PBBM to establish PCQS formed a pivotal aspect of the discussion following the presentation.

**Fig. 2:**
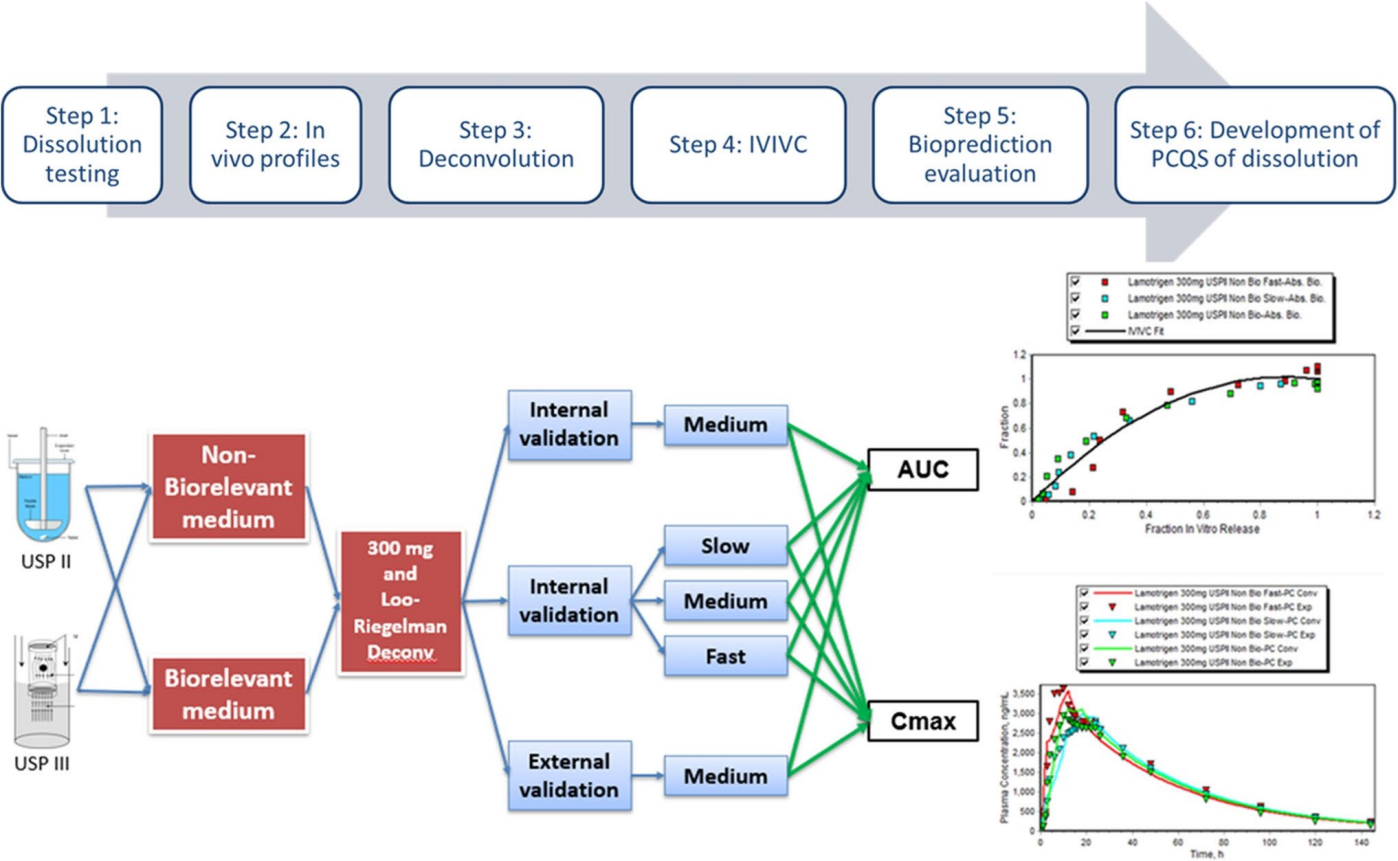
Steps for developing patient-centric quality standards (PCQS) for dissolution using in vitro-in vivo correlation (IVIVC) that passed predictability criteria for internal and external validation.

As shown in Fig. 2, the proposed systematic approach for establishing PCQS involved screening critical parameters for biorelevant dissolution testing to enhance the predictability of in-silico PBPK modeling. Notably, the presentation highlighted a case study involving a BCS Class IIb drug in a matrix-based enteric coated ER formulation. The study employed various dissolution conditions, including simulated gastric, intestinal, and colonic media without enzymes, mimicking gastric transit and pH gradients. The results indicated that the PBPK model adequately predicted the Cmax and AUC of both intravenous (IV) and oral immediate-release (IR) profiles.

Further research and future collaborations are needed on development of biorelevant dissolution methods, understanding food effects on drug absorption, and selection of deconvolution functions and mathematical treatments for establishing in vitro-in vivo correlation (IVIVC) models to develop PCQS for oral ER tablets.

### Summary of Presentation “Model Based Approaches to Establishing BE: Perspectives from the European Generic Sector” by Dr. Susana Almeida (Secretary General at International Generic and Biosimilar Medicines Association)

Model-informed drug development (MIDD) is becoming increasingly relevant for both generic and new drug development, encompassing a spectrum of sophisticated methodologies including PBPK/PBBM modeling, population pharmacokinetic (Pop-PK) modeling, and model-based BE assessment. The global harmonization of these approaches holds the promise of widening access to complex drug therapies for diverse patient populations worldwide. At present, interactions between applicants and regulatory agencies offer several opportunities in this field, challenges and potential solutions have been identified by developing companies.

Despite the growing interest in MIDD analyses, a common understanding regarding its appropriate application remains elusive within and across regulatory bodies and the pharmaceutical industry. The lack of documentation standards, model validation/assessment and uniform understanding of terminology has hindered greater utilization of MIDD approaches. This leads to over reliance on empirical approaches, inefficient drug development strategies and study designs.

Recognizing the escalating significance of MIDD, the ICH has elevated it to the status of a dedicated topic, M15, aimed at furnishing comprehensive guidance on the foundational principles of MIDD, applicable across model-based paradigms. Acknowledging the inherent disparity between the rapid pace of scientific advancements and the comparatively slower progress of regulatory frameworks, early joint international discussions among scientists and regulatory agencies will help reach a joint scientific thinking on this topic and hold the potential to foster a shared understanding of this domain. Such proactive engagement assumes paramount importance in enhancing regulatory predictability, shaping the development of regulatory standards, and paving the way for future harmonization endeavors.

The realization of MIDD's potential hinges upon overcoming several key hurdles. Efforts to establish standardized protocols for documentation, validation, and nomenclature are imperative for streamlining MIDD. Furthermore, capacity-building initiatives aimed at enhancing proficiency in MIDD methodologies are essential for fostering a conducive environment for innovation and advancement.

Embracing the transformative potential of MIDD necessitates a concerted effort from all stakeholders, transcending geographical and sectoral boundaries. By fostering a culture of collaboration, transparency, and innovation, the global community can harness the power of model-informed approaches to revolutionize drug development paradigms, ensuring timely access to safe, effective, and affordable therapies for patients worldwide.

### Summary of Presentation “PBPK/PBBM Applications in Drug Development” by Dr. Tycho Heimbach (Senior Principal Scientist/Director in Pharmaceutical Sciences, Merck Research Laboratories)

Dr. Tycho Heimbach presented two case studies related to PBPK/PBBM applications in drug development for fevipiprant and molnupiravir.

Fevipiprant is a zwitterionic, low molecular weight, BCS Class IV drug substance. PBBM was implemented to assess the impact of in vitro dissolution on the in vivo performance of IR film coated tablets during drug development and scaling-up to commercial scale [16]. A fevipiprant dissolution safe space was established using observed IV and oral PK data from BE and non-BE formulation batches. Quality control dissolution profiles with tablets were used as GastroPlus^TM^ model inputs to estimate the in vivo dissolution in the GI tract and to simulate human exposure. The PBBM performance was demonstrated for various oral dosage forms (150 ‒ 450 mg), including the non-BE batches in fasted healthy adults. To define the safe space at 450 mg, simulations were performed using theoretical, virtual dissolution profiles. A specification of Q = 80% dissolved after 60 min for an IR oral solid dosage form reflected the boundaries of the safe space.

Molnupiravir (MOV; MK-4482, EIDD-2801) is an oral antiviral that has received emergency use authorization by the FDA for the treatment of adults with mild-to-moderate COVID-19. MOV is a prodrug of N-hydroxycytidine (NHC; formerly EIDD-1931) which inhibits viral replication of SARS-CoV-2. MOV is classified as a BCS Class I compound (high solubility, high permeability). While MOV showed low permeability with the Caco-2 cell model, the rat intestinal perfusion model showed that MOV has higher permeability than metoprolol. The BE safe-space was to be established for different formulations. To characterize human MOV absorption and systemic PK of NHC and to assess the BE of batches from three manufacturing sites, PBBM was undertaken using a dissolution method as per FDA guidance document [1]. The developed models were qualified against the clinically observed results from the literature [17]. Based on PBBM developed in GastroPlus^TM^, the MOV prodrug has a high estimated absorption in human (Fa>85%). Predictive errors (PEs) were <25% for Cmax and AUC. A NHC PK BE safe space was established using oral solution and capsule data. Due to the high equilibrium solubility of MOV, and rapid generation of NHC in vivo, minor changes in MOV dissolution do not impact NHC systemic PKs. For the dissolution model, the Z-factors from in vitro data were used for the simulations by setting the dissolution model as Z-factor model. The proposed PBBM, along with appropriate MOV dissolution data, is suitable for applications to support potential future post-approval formulation changes.

### Summary of Presentation “Integration of Biopredictive Dissolution and PBPK Models for Evaluation of GI Locally Acting Products (Part 1)” by Dr. Sherin Thomas (Pharmacologist in DQMM, ORS, OGD of the FDA)

Dr. Thomas presented on using oral PBPK model as a risk assessment tool of BE for GI locally acting drug products. The presentation focused on a case study of a GI locally acting product obtained from an ANDA submission. The drug product was a mesalamine delayed release (DR) tablet with the colon as the site of action. The product-specific guidance (PSG) for mesalamine DR tablets recommends a fasting PK BE study, a fed PK BE study, and comparative three-stage dissolution studies with stage 3 at four different pHs (6.5, 6.8, 7.2, and 7.5) [18]. For this case example, dissolution studies showed differences between Test and RLD products with similarity factors (*f*2) less than 50 at pH 6.5 and 6.8 buffer conditions for stage 3 of dissolution testing. During ANDA assessment, a PBPK absorption model was developed and used to evaluate the risk of bioinequivalence for the Test product at the site of action (i.e., colon).

**Fig. 3:**
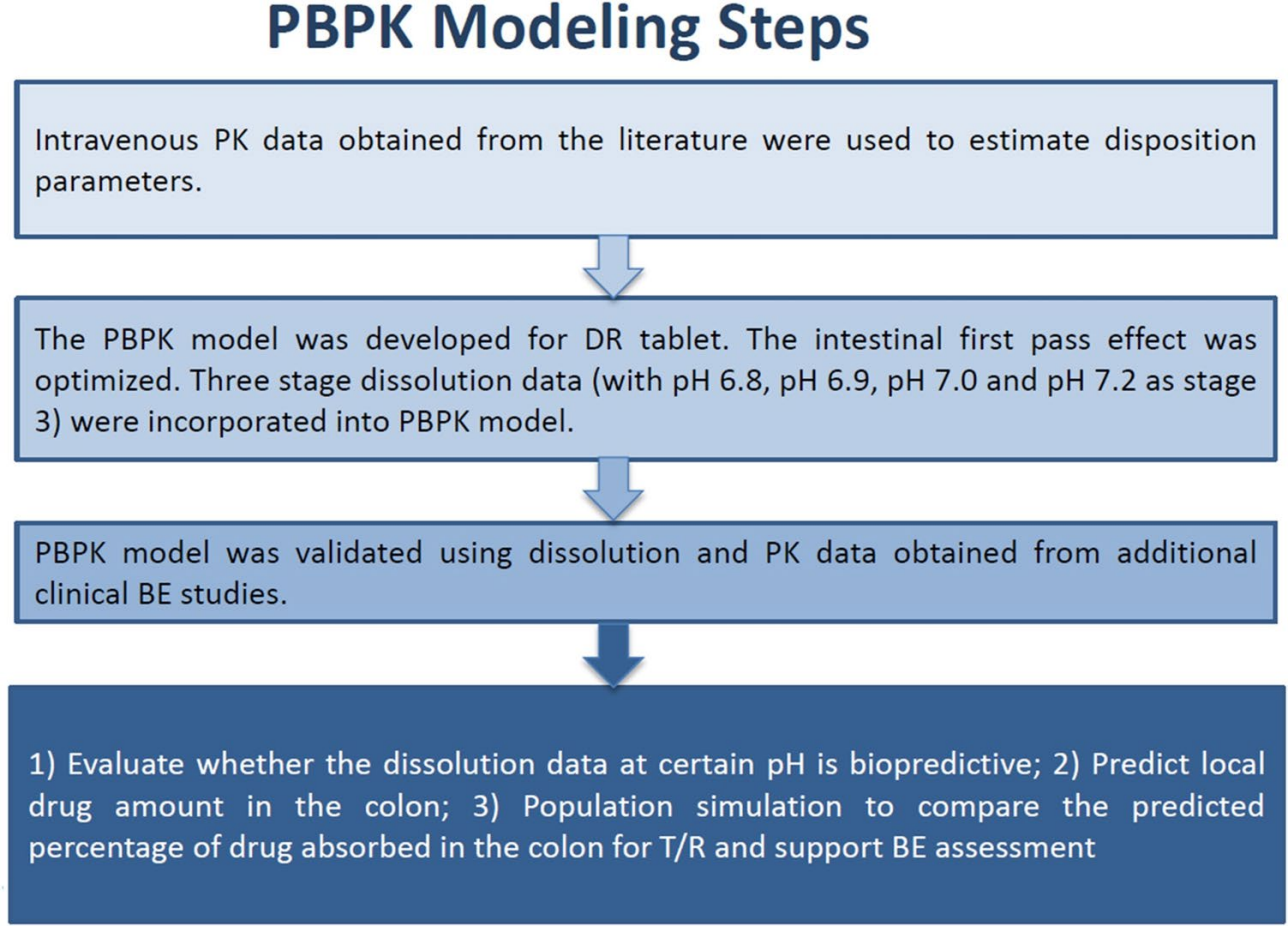
PBPK modeling workflow for the gastrointestinal (GI) locally acting drug product, Mesalamine delayed-release (DR) tablet.

The PBPK modeling workflow was shown in Fig. 3. Specifically, IV PK data obtained from the literature were used to estimate disposition parameters. Three-stage dissolution data with stage 1 in 0.1N HCl for 2 hours, stage 2 in pH 6.4 phosphate buffer for 1 hour and stage 3 in pH 7.2 phosphate buffer for 8 hours was incorporated to inform the absorption parameters. pH-dependent solubility data were incorporated into the developed model. The intestinal transit time was optimized to mimic most drug absorption in the colon and absence of drug absorption in stomach and early small intestine (duodenum and jejunum) since the formulation shows delayed pH-dependent drug release. The developed PBPK model was validated using dissolution and PK data obtained from additional clinical BE studies. The validated model was then used to evaluate whether the three-stage dissolution data with stage 3 at multiple pHs is biopredictive of PK profiles (i.e., PE<25% for Cmax and AUC). VBE simulation was conducted to compare percentage of drug absorbed in the colon between Test product and RLD to support BE assessment. Simulation results showed that dissolution profile for stage 3 at pH 6.8 was not biopredictive for the systemic exposure of Test product with PE for Cmax and AUC of ≥30%. In contrast, the predicted systemic exposure and amount of mesalamine in colon was found to be similar between RLD and Test product at pH 6.9 and above. Further, VBE simulations (n=25) showed that the percentage of drug absorbed in the colon is similar between RLD and Test product with the 90% CI of the T/R ratio falling within 80-125%.

The PBPK model predicted local drug amount in the colon by incorporating dissolution data at different pH conditions and supported that:

(i) The three-stage dissolution profiles at both pH 7.0 and pH 7.2 (as stage 3) may be biopredictive/biorelevant to the local and systemic exposure. Individual PK simulations were conducted using the validated PBPK model by incorporating the biopredictive dissolution data, and the simulation results appeared to cover the whole range of observed PK profiles.
(ii) The percentage of drug absorbed in the colon is similar between the RLD and Test product as predicted by the biopredictive dissolution data. 90% CI for the T/R ratio for percentage of drug absorbed in the colon was calculated based on population simulations, and it was within 80-125%.
(iii) The predicted amount of mesalamine in colon is similar between RLD and Test product using the biopredictive dissolution data.

Totality of evidence based on dissolution data, formulation differences, gut physiology considerations along with PBPK modeling results was used to conclude that the risk is low for the test product to exhibit bioinequivalence at site of action.

Currently, FDA has multiple research contracts/grants focused on improving in vitro BE methods and developing predictive in silico models. These projects intend to develop in vitro biopharmaceutic data including solubility and dissolution data generated for GI locally acting drug products and use the generated in vitro biopharmaceutics data to conduct model-based VBE evaluation in healthy and patient population.

### Summary of Presentation “Integration of Biopredictive Dissolution and PBPK Models for Evaluation of GI Locally Acting Products (Part 2)” by Prof. Nikoletta Fotaki (Professor of Biopharmaceutics, Centre for Therapeutic Innovation, University of Bath)

Prof. Nikoletta Fotaki presented the impact of GI disease on oral drug absorption, in terms of transit times and hydrodynamics, the composition of GI fluids, permeability, metabolic enzymes and the microbiota. She then described the development of media simulating the GI conditions in patients with Crohn’s disease (CD) using a design of experiment approach based on identified differences between CD patients and healthy subjects. The increased prevalence of bile acid malabsorption, the reduced gastric acid secretion and the increased fecal osmolality observed in CD patients were reflected in the CD biorelevant media. Based on solubility studies of a wide range of compounds in terms of their physicochemical properties in healthy and the disease state biorelevant media, the risk of altered solubilisation in patients with CD was assessed.

**Fig. 4:**
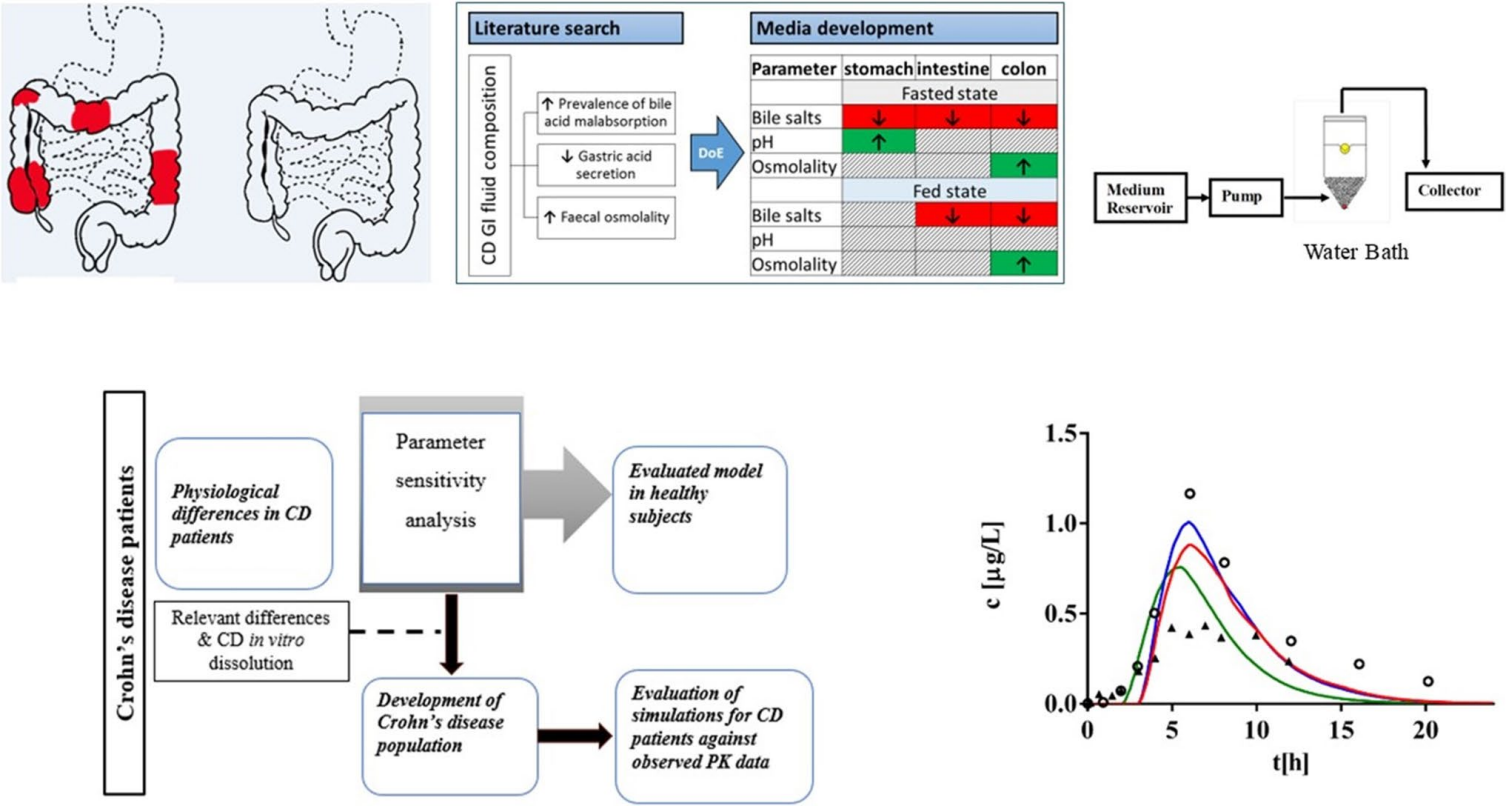
PBPK modeling framework for evaluating the impact of GI disease on oral drug absorption in Crohn’s disease (CD) patients.

Afterwards, Prof. Fotaki presented the prediction of oral absorption in CD patients based on a PBPK modeling approach with integrated biorelevant solubility and dissolution data (Fig. 4). The steps of this approach were shown based on a case study for a controlled-release (CR) formulation of budesonide [19]. Firstly, solubility studies in healthy and CD state biorelevant media and drug release studies in these media using a flow through cell method reflecting the differences in hydrodynamics between healthy and CD states were performed. Then a PBPK model reflecting the healthy state was developed and the method of input of the dissolution data was investigated. Afterwards, a PBPK model reflecting the disease state was developed and the investigated parameters included the CYP3A4 activity, the plasma proteins, the GI transit time and the gastric pH based on a parameter sensitivity analysis (PSA). The CD population designed in the model reflected these factors. After integration of the CD release studies a successful prediction after the administration of the budesonide CR formulation in CD patients was obtained. She concluded her presentation emphasizing that the pathophysiological changes in CD patients may alter the properties of the GI fluids and the alteration of solubility of poorly soluble compounds in CD simulated GI fluids could reveal implications for absorption in patients with CD. Successful predictions for oral product performance in CD patients can be achieved with biopharmaceutics tools (biorelevant solubility and release/dissolution tests integrated in PBPK models where pathophysiological differences in CD patients are considered). Full validation of these models can be achieved with investigation of several drugs and formulations. PBPK models could indicate when GI diseases pose a risk for safety and efficacy and dose adjustments are needed.

### Summary of Presentation “PBPK Models for the Evaluation of GI Locally Acting Products, from Industry Perspectives” by Harshil Shah (Senior Manager in Bioequivalence, Cosette Pharmaceuticals Inc.)

PBPK modeling finds diverse applications for both new and generic drug development (Table I). Specifically, PBPK model applications for new drug development include dosing optimization, formulation development, assessing food effects, pediatric studies, drug-drug interactions (DDIs), BE assessments, and route of administration selection. On the other hand, it plays a crucial role in generic drug development, such as aiding in pre-clinical to clinical BE extrapolation, evaluating clinical impact, proposing biowaiver of lower strength, assessing BE between Test and RLD product following regulatory guidelines.

**Table I:** PBPK Modeling Applications for new and Generic Drug Development

New Drug Development	Generic Drug Development
Dosing optimization	Pre-clinical to clinical BE extrapolation
Formulation development	Evaluating clinical impact
Food effects assessment	Biowaiver of lower strength
Pediatric studies	BE assessment between Test and RLD product
Drug-drug interactions (DDIs)	
Bioequivalence (BE) assessments	
Route of administration selection	

**Fig. 5:**
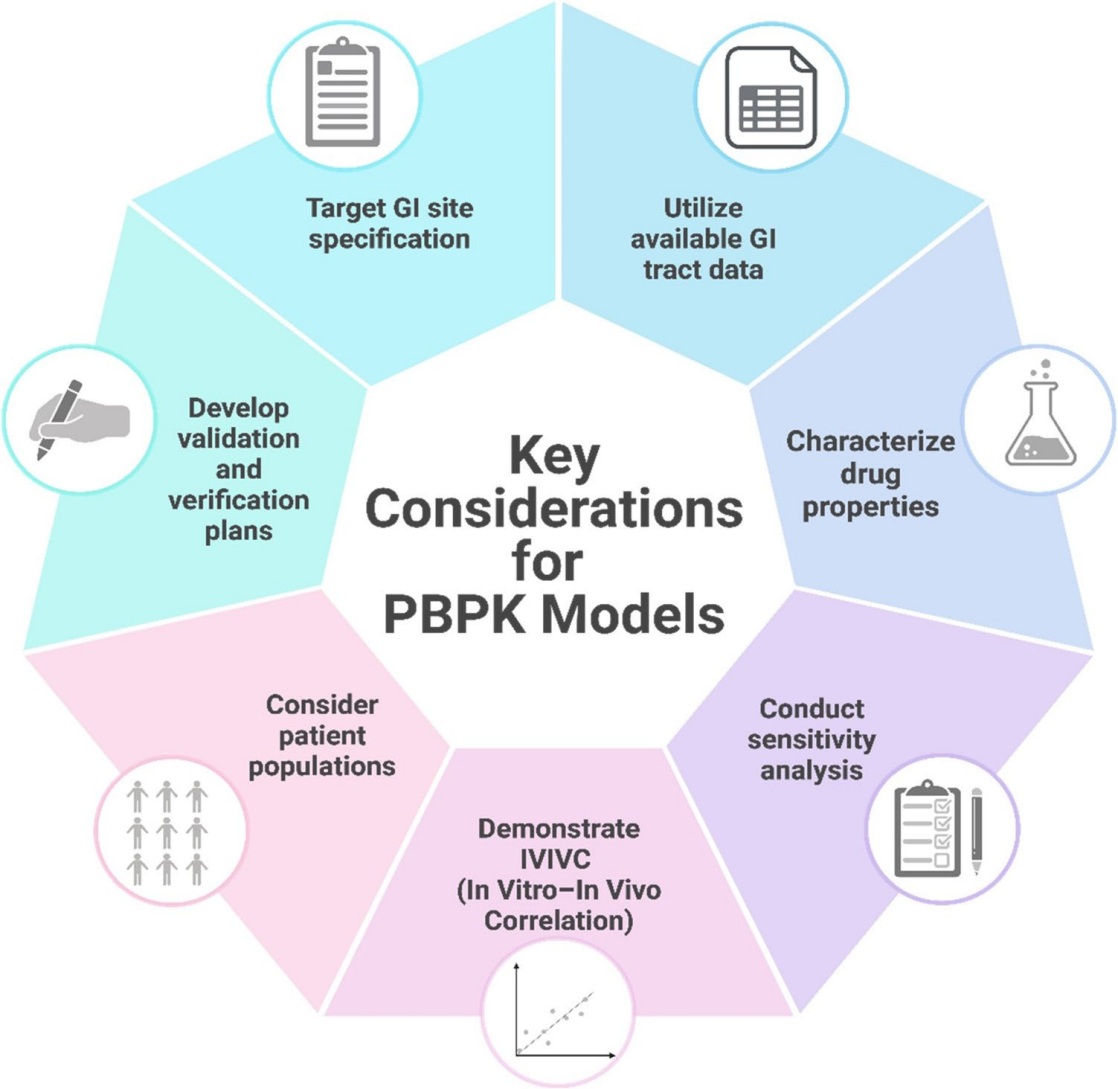
Key considerations for PBPK model development, validation, and application.

For GI locally acting products, PBPK modeling is crucial. These products undergo dissolution in the GI tract, providing drug availability at the site of action. BE studies using systemic concentration-time profiles may be insensitive to tease out differences that occur locally, necessitating conduct of studies with pharmacodynamic end-points that require greater subject numbers. In vitro dissolution testing is vital for identifying the impact of formulation differences. PBPK models that integrate physiological and drug property knowledge can predict potential PK differences in virtual populations. Several challenges exist in developing GI locally acting products, including precise drug delivery at target site, variability in GI physiology, inherent complexity of the GI tract, interactions with food, dosage form challenges, in vivo testing complexities, patient variability, and resource intensity. PBPK models address challenges by incorporating anatomical and physiological variability, dividing the GI tract into compartments, simulating controlled drug release, assessing food effects, integrating in vitro/ex vivo data, and providing mechanistic understanding of drug behavior. Key considerations for PBPK models include specifying the target GI site, utilizing available data, gathering relevant GI tract data, characterizing drug properties, conducting sensitivity analysis, demonstrating IVIVC, considering patient populations, and developing a validation and verification plan (see Fig. 5).

Developing PBPK models is a multifaceted process requiring a detailed understanding of input parameters, model structure, the intended purpose of use, and thorough validation. Verifying PBPK models for GI locally acting products is key for regulatory and drug development decisions. Models failing to predict systemic PK profiles may be disqualified. PBPK models aid in assessing whether systemic drug exposure reflects local drug delivery. Examples include correlations between systemic mesalamine plasma PK and its local GI distribution. Regulatory authorities, including the FDA and European Medicines Agency (EMA), express interest in PBPK modeling's application to support drug development and decision-making.

In conclusion, PBPK modeling is a versatile tool in drug development, with specific importance in addressing challenges associated with GI locally acting products. Its ability to integrate diverse data and simulate complex physiological processes makes it an asset in optimizing drug development processes and ensuring the efficacy of GI locally acting products. Model verification for GI locally acting products is crucial for regulatory and drug development decisions, though challenging due to difficulties in obtaining drug concentrations at the site of action. Advancements in technology, generating relevant in vitro and in vivo data reflecting local drug delivery, leveraging systemic PK, and using additional data collectively form a weight-of-evidence approach for model validation.

## Panel Discussion

To explore and integrate current knowledge of recent advances in PBPK modeling for regulatory purposes and to support global harmonization, a panel discussion was conducted at the end of this session where representatives from regulatory agencies, industry, and academia were present. The panel discussion was moderated by Dr. Liang Zhao, Division Director from DQMM, ORS, OGD, CDER of the FDA, and Dr. Yu Chung Tsang, President of YCT Scientific Inc. In addition to podium speakers (Dr. Fang Wu, Dr. Muhammad Ashraf, Dr. Susana Almeida, Dr. Tycho Heimbach, Dr. Sherin Thomas, Dr. Nikoletta Fotaki, and Harshil Shah; refer to previous sessions for their affiliations), the panelists include Dr. Xiaojian Jiang (Deputy Director from the Divisions of Bioequivalence II (DB II), Office of Bioequivalence (OB), OGD, CDER, FDA), Dr. Rebecca Moody (Pharmaceutical Scientist from the Immediate Office (IO), Office of Pharmaceutical Quality Assessment II (OPQA II), OPQ, FDA), Dr. Amin Rostami‐Hodjegan (Professor of Systems Pharmacology, University of Manchester), and Dr. Romi Singh (Senior Vice President in Sun Pharmaceuticals, India). During the panel, both prepared questions and questions from the audience were discussed and addressed. The panelists' comments are summarized below:

### Question 1 - Based on Your Experiences, what are the Latest Advances and Utilities of using oral PBPK Absorption Modeling to Support Generic or New Drug Regulatory Submissions?

The panel provided the following case examples of utilizing oral PBPK absorption modeling to support generic or new drug regulatory submissions: (i) to support drug quality development; (ii) to set clinically relevant dissolution or particle size distribution (PSD) specifications; (iii) to assess the impact of non-comparable in vitro ADD studies across different dose strengths on systemic exposure and BE; (iv) to justify for BE study design using subjects of a single sex as the study population; (v) to evaluate BE for GI locally acting drug products; (vi) to incorporate inter- and intra-subject variability of parameters in the PBPK modeling and VBE trials; and (vii) to address BE related questions, such as the relevance of apparent Tmax and Tlag differences, impact of non-comparable in vitro dissolution profiles between Test and RLD or reference standard products on BE results, or justification to request biowaiver for non-proportional lower strength products. It was noted from the regulatory assessor’s perspective, there is concern about sufficient validation of the applicant’s submitted PBPK model.

### Question 2 - What are the Challenges and Possible Solutions for Developing Patient-centric Dissolution Quality Standards using PBPK Modeling?

The discussion focused on two main topics: the challenges and potential solutions for developing patient-centric dissolution quality standards by utilizing PBPK modeling approaches. Panelists from regulatory agencies initiated the discussion by outlining the current challenges in developing patient-centric dissolution specifications based on PBPK absorption modeling. These challenges include:

- The struggle to mimic in vivo drug release profiles using in vitro dissolution methods with available apparatuses that can account for food effects or different disease conditions.
- Incomplete information from clinical studies regarding food effects and disease states during PBPK model development.
- Difficulty in establishing a clinically relevant in vitro-in vivo link when developing patient-centric dissolution specifications due to insufficient clinical studies with PK or formulation variants.
- Different dissolution data inputs in QC medium and/or physiologically relevant media.
- Lack of global acceptance and harmonization and minimal transparency between industries and regulatory agencies regarding data sharing expectations.

To address these challenges, the panelists first identified key principles for developing patient-centric dissolution quality standards and then proposed solutions. For example, they recommended combining multiple dissolution studies at different pH ranges to develop patient-centric dissolution quality standards rather than relying on studies conducted at a single pH. This is because the pH, shear stress, and bile salts are continuously changing in the GI tract. The panel further stated that no single dissolution method can capture all the physiological conditions necessary to be biopredictive of all scenarios of PK data; it is impossible to replicate every aspect of an in vivo situation in an in vitro setup. However, PBBM along with PSA can be utilized as a screening tool to identify the critical quality attributes (CQAs) of formulations, provide a mechanistic understanding of the in vivo situation, and enable refinement of formulation and dissolution methods. Given the complexity of the GI tract, sometimes a simple dissolution method (e.g., USP II paddle method) will suffice, but in other situations a more complex method (e.g., USP IV flow-through-cell method) may work better. Another ultimate solution might be hosting more workshops like this one to encourage early communication between industries and regulatory agencies regarding the feedback on using PBPK modeling approaches to establish an in vitro-in vivo link, rather than waiting until specifications fail in QC media.

In addition to discussing challenges and potential solutions, the panelists from various industries deliberated on the current state of routine PBPK modeling utilities during the drug product life cycle, sharing successful case examples of regulatory submission and approval. Representatives from innovator companies specified that only a small number of PBBM or PBPK model applications reach regulatory agencies due to strategic decisions made during drug development. For instance, when a company decided to develop a modified-release (MR) product early in the drug development stage, they would evaluate all available models to select the ideal candidate for the MR formulation. Similarly, when dealing with oncology drugs with poor water solubility, PBBM or PBPK models would be used to inform decision-making about whether a costly enabled formulation is necessary. For oral drug product development, PBPK modeling is applied for almost every oral project, with very few exceptions, such as formulation decisions or prospective DDI assessments. A panelist with major pharma experience indicated that there was only one successful case example out of three submissions that got approval involving level A IVIVC using PBPK modeling. In contrast, generic companies face different considerations. Due to the nature of generic drug development, where the goal is to replicate an already approved product, the application of PBPK modeling might be less extensive. Generic companies often focus on using PBPK models, including physiologically based IVIVC, to inform routine internal oral product development and to select dissolution media. Similarly, early communications between industries and regulatory agencies through workshops and model focused meetings including pre-ANDA meetings and model-integrated evidence (MIE) pilot program regarding the feedback on using PBPK modeling approaches to establish IVIVC/IVIVR would be beneficial.

### Question 3 - When do You Think the Community Should Consider Harmonization Opportunities for oral drug products (including MR Products) using PBPK Modeling and Simulation Approach? What are these Opportunities?

The panel engaged in an in-depth discussion on the status, process, and opportunities for global harmonization of oral drug products using PBPK modeling and simulation. The ICH is renowned for its role in uniting regulatory agencies and pharmaceutical industries to address the scientific and technical aspects of drug development and registration. The panelists highlighted that harmonization efforts often commence well before formal ICH processes begin. The primary objective for the subject-matter experts (SMEs) convened at this event was to facilitate dialogue, establish foundational principles, and set the stage for future harmonization. Past experiences and ongoing efforts to initiate the M13 guideline for IR products incorporating the latest knowledge on MR products, will undoubtedly pave the way for future harmonization efforts for MR products. Lastly, the panel underscored that besides traditional methods of implementing PBPK modeling in drug development (i.e., informing routine internal formulation development and selecting dissolution media for oral drug products), we can also consider additional PBPK utilities, e.g., using PBPK model to support demonstration of BE for non-biostrengths for MR products. This can support further development of ICH guidelines.

### Question 4 - What are the Key Considerations for Adequately Developing/Validating PBPK Modeling for Local Drug Exposure in the GI tract of GI Locally Acting Products in Healthy vs. Patient Population?

During the final part of the panel discussion, experts from regulatory agencies, academia, and the pharmaceutical industry discussed the critical factors for adequately developing and validating PBPK models for predicting local drug exposure of GI locally acting drug products in healthy subjects versus patients. The panelists emphasized the importance of determining and selecting the most biopredictive/biorelevant dissolution conditions as model inputs to accurately reflect the systemic exposures of GI locally acting drugs and to predict local BA in both healthy and diseased populations. These factors become more critical when considering patient populations, as the developed absorption PBPK models must account for physiological and biological changes under different disease states, such as variations in pH conditions, microbiome, and fluid composition in the GI tract, as well as abundance of enzymes and transporters, especially for those drugs with first-pass metabolism (e.g., in gut wall or gut lumen) and efflux transporters (e.g., BCRP and P-gp) involved absorption. The panel also underscored the challenges in validating local drug exposure and highlighted the necessity of extrapolating the developed PBPK models from animals to humans when local concentration data are only available from animal studies. In addition to discussing challenges and critical aspects of model development and validation, the panel explored potential opportunities for GI locally acting drug products. For instance, addressing the variability of patient populations could help validate the developed PBPK absorption models. The panel recommended implementing PSA throughout the PBPK model development and validation processes to demonstrate model validity and the capability of predicting IVIVC. However, PSA, especially local sensitivity analysis, should be applied with caution to ensure that correlations between physiological parameters in the GI tract are carefully considered. Despite the fundamental challenge that the developed model cannot be fully validated due to a lack of human clinical studies with measured local concentrations, it can be indirectly validated later on. Researchers and regulators in related fields should continue to advance the model, use PBPK modeling and simulation results to fill knowledge gaps by integrating ambiguous dissolution and PK data, and later apply real-world evidence to support their decisions.

## Roundtable Discussion

Following the hybrid (virtual and in-person) sessions, a dedicated in-person roundtable discussion session engaged all the attendees from regulatory agencies, industry, and academia to discuss in a broader and deeper scope based on their experience. This session focused on the discussion of current perspectives, knowledge gaps, as well as future harmonization opportunities on utilities of PBPK absorption modeling and VBE simulation in supporting global regulatory submissions of oral drug products (e.g., IR and MR/CR drug products). This session was moderated by Myong‐Jin Kim, PhD (Director for Division of Therapeutic Performance II, ORS, OGD, FDA), Yu Chung Tsang, PhD (President, YCT Scientific Inc.), and Nikunjkumar Patel, PhD (Senior Director of PBPK Consultancy, Certara Inc.). The participants discussed and addressed key deliberations involving the utilization of biopredictive/biorelevant oral PBPK models to support innovator and generic drug development and submission. In the following part of this report, we have summarized the case studies presented by Dr. Tsang and Dr. Patel as well as three critical questions developed a priori and discussed during this roundtable discussion.

The discussion started with a brief presentation by two of the co-moderators. Dr. Tsang presented a common application of PBPK modeling and simulation by generic industry for the development of clinically relevant dissolution specifications. Experience of this application for a CR product was shared in the presentation. By applying PBPK modeling using in-vitro dissolution data and in-vivo BA data, dissolution boundaries that provide PK profiles within BE limits with respect to the reference product were established. With the established dissolution specification limits, biowaiver could be granted if future batches of the CR product meet the limits.

Dr. Patel followed by presenting the opportunities for PBPK modeling in supporting BCS Class IIa and Class III drugs with examples from scientific literature. Weakly acidic BCS Class II drugs with pKa ≤5, also termed as BCS Class IIa, have sufficient time for complete dissolution and absorption in small intestine due to high solubility at intestinal pH range [20]. In such cases, biowaivers may be scientifically sound, if relevant dissolution test standards are met. PBPK modeling can help provide clinically relevant extrapolation of in vitro dissolution data, solubility and permeability interplay using simulated human physiological environment. This has been nicely exemplified by a case study of bempedoic acid using PBPK modeling and VBE simulation approach within Simcyp simulator, where the authors have used mechanistic modeling for formulation characterization and in vitro dissolution of suspension versus tablet formulations [21]. Federation International Pharmaceutique (FIP) in their concept paper on the biowaiver monographs stated that there seems to be a little risk associated with applying biowaiver to weak acids with adequate solubility at pH 6.8 and above based on the current evidence [22]. Biowaiver monographs also exist in scientific literature for BCS Class IIa drugs [23]. Sufficiently validated PBPK model along with in vitro dissolution comparisons, clinical evidence of absorption (hADME study, absolute BA study, literature evidence), similarity in formulation compositions or lack of solubility/permeability influencing excipients can potentially be used as totality of evidence to support biowaivers for IR formulations of BCS Class IIa drugs. Sensitivity analysis using validated PBPK models can also help assess product quality risk and establish clinically relevant safe space.

Dr. Patel then discussed about utility of PBPK modeling in supporting BCS Class III drug products for which he cited a publication titled “Scientific considerations to move towards biowaiver for BCS Class III drugs: How modeling and simulation can help” [24]. This publication discusses how PBPK modeling could potentially help assess BE between non-Q1/Q2 oral IR products of BCS Class III drugs. Dr. Patel also supported this with an example of elagolix where widening of the dissolution specifications was supported by MIE approach using Simcyp simulator [25]. Furthermore, PBPK modeling and VBE simulation within Simcyp simulator was conducted to evaluate BE between the tablet and capsule formulations of elagolix in support of a regulatory biowaiver [26]. With these scientific case studies and literature, Dr. Patel posed questions for expanded roundtable discussions on how to utilize PBPK models to build MIE for such drug products containing BCS Class IIa and III drugs and if there is a possibility of harmonization across regulatory agencies.

### Question 1 - Based on Your Experiences, what are the Latest Advances and Utilities of using Oral PBPK Absorption Modeling to Support Generic or New Drug Regulatory Submissions?

Experts from both innovator and generic drug industry shared their experiences and/or perspectives related to recent advances and utilities of oral PBPK absorption modeling to support regulatory submissions. In addition to those discussed during panel discussion, innovator drug companies have utilized PBPK modeling to substantiate post-approval formulation changes based on in vitro study results, to support BCS Class III biowaiver proposal to some extent, and to help human dose selection and optimization from animal PK study. PBPK modeling can also be implemented to study the impact of PSD, solubility, and permeability on BA to determine the appropriate dosage form and maximum dose before conducting first-in-human (FIH) studies. As for oncology drugs, PBPK models play a crucial role in clinical study protocol development by addressing challenges related to appropriate dosing strategies and criteria for patient inclusion/exclusion with underlying DDIs, including interactions with proton pump inhibitors (PPIs). In addition, PBPK modeling can potentially bridge between phase II and phase III studies to account for interindividual variability, dose selection, food effect when changing from capsules to tablets, and efficiency while scaling up manufacturing processes (e.g., scale-up and post-approval changes, SUPAC). Overall, oral PBPK modeling is widely utilized by innovator drug companies.

Following the viewpoints shared by the representatives from innovator industry, representatives from generic drug companies indicated that utilities of PBPK modeling have not been fully embraced partly due to challenges related to uncertainty of expectation for acceptance by regulatory bodies (e.g., FDA, EMA, and Health Canada), for example, requirement for sufficient validation of the developed oral PBPK model. Nevertheless, even with current challenges of utilizing PBPK absorption modeling in regulatory submissions, generic industry still uses PBPK modeling to establish dissolution specifications and safe space to support generic drug approval. Moreover, PBPK modeling can provide deeper insights to develop alternative BE study design with regard to required sample size or study duration, especially for complex drug products, including long-acting injectables. Lastly, PBPK modeling has been applied for supporting waivers of in vivo BE studies by generic drug companies.

### Question 2 - What are the Harmonization Opportunities in Terms of using PBPK Absorption Modeling for Regulatory Submissions?

The discussion began with the insights from the attendees regarding harmonization opportunities and their challenges observed within or across different institutions/regulatory bodies related to applying PBPK absorption modeling for regulatory submissions. Acknowledging the barriers, including limited experiences and resources to review regulatory submissions involving application of PBPK modeling, participants suggested that data and experience sharing within and between institutions/regulatory agencies, for example, model master files (MMFs) and cluster groups may enormously benefit internal and global harmonization. Specifically, well-developed and sufficiently validated MMFs play a critical role on possibly supporting biowaivers of clinical comparative studies, including fed BE study. With this context, regulatory agencies are encouraged to publish guidance and successful case studies for applicants to follow, likewise, applicants are encouraged to share relevant data to facilitate regulatory harmonization. In addition, the discussion underscored the importance of communication among model developers, industry, and regulatory agencies to carefully develop a strategic plan for implementation of MMFs as well as data sharing.

### Question 3 - What are the key considerations for adequately developing/validating PBPK modeling for local drug exposure in the GI tract of GI locally acting products?

The final question surrounded the key deliberations to adequately develop and validate the PBPK model for oral locally acting drugs in the GI tract. Specifically, the discussion emphasized the need to reconsider the suitability for applying current criteria of *f*2 to GI locally acting drugs. With this regard, attendees mostly reached consensus that real case examples may be beneficial for reevaluating current *f*2 criteria. However, due to unbalanced large number of positive case examples over negative case examples, which may not be reflective of the real situations, hypothetical formulations with theoretical dissolution profiles may be considered. Participants with pertinent expertise further underscored the evolving realization that while a developed PBPK model may accurately predict drug concentrations in systemic circulation, it might not exhibit the same predictive capability for local drug exposure. In contrary to the assumption that one dissolution method can be biopredictive of systemic drug concentrations following administration of oral drug products, dissolution profiles obtained at different pH may be needed to predict local concentrations for GI locally acting drugs. Overall, additional research on biopredictive dissolution and PBPK modeling for GI locally acting products and sharing case examples through publications are useful and encouraged for further understanding of using in vitro testing data to predict local BA of these drug products.

## Conclusions

This session of the workshop shared the latest advances of PBPK modeling in regulatory contexts as well as the current thinking regarding opportunities to achieve global harmonization for BE assessment of oral drug products. Combining multiple dissolution studies at different pH ranges can aid in developing patient-centric dissolution specifications. PBPK/PBBM modeling, along with PSA, can facilitate the refinement of formulations and dissolution methods. Additionally, early communication between industries and regulatory agencies is encouraged to obtain feedback on using PBPK modeling approaches to establish an in vitro-in vivo link when developing patient-centric dissolution quality standards. Specifically, for GI locally acting drug products, one of the major challenges is the inability to validate the PBPK model for local BA. These models can be validated for systemic drug concentrations and can add value towards making decisions for risk-based BE assessment. The modeling and simulations results should align with the mechanistic understanding of the drug product. A totality of evidence-based approach taking account of all available data in addition to PBPK modeling based-evidence should be considered. Moving forward, to advance both internal and international harmonization, it is crucial to promote global collaboration and research by sharing knowledge and experiences through publishing guidelines, successful case studies, hosting public workshops, and submitting well-established and sufficiently validated PBPK/PBBM models, including MMF.

## Declarations

### Disclaimers

The views expressed in this article are those of the authors and should not be construed to represent the FDA’s views or policies. The mention of commercial products, their sources, or their use in connection with material reported herein is not to be construed as either an actual or implied endorsement of such products by the FDA.
